# Sharing Medical Data for Health Research: The Early Personal Health Record Experience

**DOI:** 10.2196/jmir.1356

**Published:** 2010-05-25

**Authors:** Elissa R Weitzman, Liljana Kaci, Kenneth D Mandl

**Affiliations:** ^5^Manton Center for Orphan DiseaseChildren's Hospital BostonBostonUnited States; ^4^Division of Emergency MedicineChildren's Hospital BostonBostonUnited States; ^3^Children's Hospital Informatics Program at the Harvard-MIT Division of Health Sciences and TechnologyChildren's Hospital BostonBostonUnited States; ^2^Division of Adolescent MedicineChildren's HospitalBostonUnited States; ^1^Department of PediatricsHarvard Medical SchoolBostonUnited States

**Keywords:** Medical records, personally controlled health records (PCHR), personal health records, data sharing, information altruism, HITECH, public health informatics

## Abstract

**Background:**

Engaging consumers in sharing information from personally controlled health records (PCHRs) for health research may promote goals of improving care and advancing public health consistent with the federal Health Information Technology for Economic and Clinical Health (HITECH) Act. Understanding consumer willingness to share data is critical to advancing this model.

**Objective:**

The objective was to characterize consumer willingness to share PCHR data for health research and the conditions and contexts bearing on willingness to share.

**Methods:**

A mixed method approach integrating survey and narrative data was used. Survey data were collected about attitudes toward sharing PCHR information for health research from early adopters (n = 151) of a live PCHR populated with medical records and self-reported behavioral and social data. Data were analyzed using descriptive statistics and logistic regression to characterize willingness, conditions for sharing, and variations by sociodemographic factors. Narrative data were collected through semistructured focus group and one-on-one interviews with a separate sample of community members (n = 30) following exposure to PCHR demonstrations. Two independent analysts coded narrative data for major and minor themes using a shared rubric of *a priori* defined codes and an iterative inductive process. Findings were triangulated with survey results to identify patterns.

**Results:**

Of PHCR users, 138 out of 151 (91%) were willing to share medical information for health research with 89 (59%) favoring an opt-in sharing model. Willingness to share was conditioned by anonymity, research use, engagement with a trusted intermediary, transparency around PCHR access and use, and payment. Consumer-determined restrictions on content and timing of sharing may be prerequisites to sharing. Select differences in support for sharing under different conditions were observed across social groups. No gender differences were observed; however differences in age, role, and self-rated health were found. For example, students were more likely than nonstudents to favor an opt-out sharing default (unadjusted odds ratio [OR] = 2.89, 95% confidence interval [CI] 1.10 - 7.62, P  = .03). Participants over age 50 were less likely than younger participants to report that payment would increase willingness to share (unadjusted OR = 0.94, 95% CI 0.91 - 0.96, P < .001). Students were more likely than nonstudents to report that payment would increase their willingness to share (unadjusted OR 9.62, 95% CI 3.44 - 26.87, P < .001). Experiencing a public health emergency may increase willingness to share especially among persons over 50 (unadjusted OR 1.03, 95% CI 1.01 - 1.05, P = .02); however, students were less likely than non-students to report this attitude (unadjusted OR 0.13, 95% CI 0.05 - 0.36, P < .001). Finally, subjects with fair or poor self-rated health were less likely than those with good to excellent self-rated health to report that willingness to share would increase during a public health emergency (unadjusted OR 0.61, 95% CI 0.38 - 0.97, P = .04).

**Conclusions:**

Strong support for sharing of PCHR information for health research existed among early adopters and focus group participants, with support varying by social group under different conditions and contexts. Allowing users to select their preferred conditions for sharing may be vital to supporting sharing and fostering trust as may be development of safety monitoring mechanisms.

## Introduction

In the evolving landscape of health information technologies, an opportunity exists to deploy personally controlled health records (PCHR), a special category of personal health record, as a platform for engaging consumers in public health research. The PCHR technology is designed to enable this. The platform model of the PCHR has three key properties [1]. First, data across sites of care are integrated into a repository leveraging the patient’s rights to those data. This is achieved in a manner very similar to the way a consumer might use the financial software Quicken (or the newer web application, Mint.com) to aggregate personal financial data across multiple sources. The second property is that the data are under strict personal control. The PCHR users “own” this copy of their medical data and can choose to share it with care providers, family members, or other software applications. The third property is that third party applications may connect to the PCHR central data repository across a standard application programming interface, much as applications from the iPhone apps store can be connected to the iPhone platform. Whether consumers are willing to engage with applications that support sharing their data with public health—which may be an important alternative to extracting patient data *en masse* from electronic health records—is a crucial question.

Broadly diffused personally controlled health records (PCHRs) may serve as uniquely rich consumer-centered environments through which to engage cohorts in consented public health research. This recently articulated vision [1,2] is aligned with the newly enacted Health Information Technology for Economic and Clinical Health (HITECH) Act [3]. HITECH, through a US $24 billion appropriation, aims to “harness the full potential of digital technology to prevent and treat illnesses and to improve health” through providing high quality information to providers for care improvement and through simplifying “collection, aggregation, and analysis of anonymized health information” for public health and safety.

Subscription models for PCHRs, enabling the consumer to add data sources from diverse sites such as clinics, hospitals, pharmacies, and labs, afford the technical means for integrating streams of institutionally tethered health information into a master, patient controlled record that affords views of health and service domains [4,5]. Annotation and possibly even survey features of PCHRs allow for capture of phenomenological, behavioral, and social factors that are not typically included in clinical and administrative information systems [6]. These factors in combination with clinical and biological information may help explain variation in risk, treatment, and outcome for even highly heritable diseases—an area of active research [7-10]. Integration of information that is currently missing from record systems or “siloed” in research datasets and uncoupled from clinical measures may foster improved understanding of health outcomes by supporting assessment of barriers to care, factors related to adherence, patterns of follow-up, follow-through, and adverse events.

At the population level, aggregates of such integrated and longitudinal records in a system that allows investigators to maintain ties to individual record holders may greatly advance opportunities for consented public health research and, importantly, for translation of findings to practice. Conceptual models for engaging cohorts of consumers who make their health information available for research out of altruistic or opportunistic impulses with the possibility of obtaining feedback including through participating in longitudinal research have been proposed [2]. If actualized as practice and adopted by cohorts of consumers, such solutions may contribute greatly toward closing the gap between research and practice, providing opportunity to stage and implement consented interventions along with evaluations of these interventions. Thus, both personalized medicine and public health may benefit from engaging cohorts of information altruists who share their clinical, phenotypic, and even genetic information in a PCHR-enabled model that allows for feedback and follow-up.

To move this model forward, better understanding of attitudes and willingness to engage in public health research is needed. A recent Canadian study assessed attitudes toward consent for sharing personal health information from medical records under different research scenarios and found generally favorable views among chronically ill and general population samples [11], findings that are consistent with large-sample studies conducted in other industrialized nations [12]. However, support for sharing medical record data diminished where suggested uses included commercial, profit, and marketing applications [11,13]. These findings were consistent with those reported from surveys in New Zealand, which found that patients prioritized personal control and strict restrictions on secondary data use as prerequisites for sharing medical record data for research [14]. The emergence of highly active virtual communities of persons affected by chronic or progressive illness, who share their personal health information in hopes of accelerating prevention, treatment, and cure [15,16], is evidence of the perceived value of peer-based sharing models and an indicator of the potential traction of a PCHR-based public health research model. Uncertainty and variability of opinion around appropriate consent mechanisms for use of medical record data in health research characterizes the views of research ethics boards [17-19] and patient populations [20], leaving open questions of fit between extant oversight mechanisms governing health research and the rapidly evolving information technology and research landscape associated with PCHRs [21-23].

Benefits of PCHR-enabled research models may include reductions in cost and turnaround time for the collection and application to practice of research data. Traditional research models that rely on complex methods for outreach, promotion, sampling, and collection of data provide high levels of validity and reliability at what may be prohibitive cost. In an era of resource constraint, it is crucial to develop nimble and cost-efficient approaches for engaging subjects in health research using approaches that may close the gap between researcher and subject.

The purpose of this study was to investigate willingness to share information contained in a PCHR for use in public health monitoring and research. Little is known about individuals’ attitudes toward sharing personal health information with public health agencies through this new modality and the ways that different conditions and contexts may affect attitudes among different stakeholder groups. Because deployment and diffusion of PCHRs are rare, there has been limited opportunity to investigate willingness to share health data among individuals with experience of demonstration or live PCHR systems including live systems populated with their medical record data. Understanding willingness to share and the conditions and contexts bearing on that willingness is vital to building usable, not just imagined, systems.

## Methods

### Overview

Information about attitudes toward sharing information from a PCHR with public health agencies was collected through self-report surveys administered on a PCHR platform and though focus group and one-on-one discussions with community members.

### Setting

Research activities were undertaken in an urban area within the northeastern region of the United States.

### Study Samples and Data Collection

Questions about willingness to share personal information with public health agencies for monitoring and research and about the conditions and contexts affecting willingness to share were asked of subjects from three participant groups representing varying degrees of exposure to PCHR technology using self-report survey or qualitative interview methods.

Surveys were administered prospectively on the PCHR platform to an early adopter sample of PCHR users affiliated with a local university health center who completed exit surveys after participating in a PCHR-based health promotion demonstration The demonstration exposed them to the live system populated with their own personal health information (PHI) for a nine-month period. During that time they could log in to their PCHR, view their health and medical record information, and review, complete, and save surveys in their PCHR. During the demonstration, users were sent a message informing them that they could provide others with access to their PCHR electronically by using the sharing feature in the system. The survey was administered on the PCHR platform at the demonstration’s close and it included fixed-choice and Likert-scaled items asking about health beliefs, behaviors, and attitudes toward sharing from the PCHR for public health research. The exit survey was completed by 151 of 247 (61%) of the demonstration participants. Information about sociodemographic and health characteristics of demonstration participants completing surveys was obtained using standardized self-report measures with fixed choice multi-categorical formats that were included in the surveys, as described elsewhere [24].

Qualitative data were collected using a structured protocol from PCHR usability testers (n = 13) recruited from local area worksites. Subjects were interviewed following a PCHR demonstration session in which they interacted with a live system that was not populated with their own health information.

Qualitative data were also collected through focus groups conducted with community members (n = 17) recruited from an area retiree and health advocacy mailing list. Subjects were interviewed following a demonstration of the PHCR system.

Participants in all three groups were volunteers, spoke English, and provided written informed consent and Health Insurance Portability and Accountability Act (HIPAA) authorization for sharing personal health information when piloting or evaluating live records populated with their personal health information. Research was reviewed and approved by the Children’s Hospital Institutional Review Board. The involvement in survey research of participants from the live demonstration was reviewed by the demonstration site local IRB as well.

### Analytic Approach

Survey data for the first study group of demonstration evaluation participants were extracted from participants’ PCHRs and exported to a SAS file for analysis (SAS version 9.2, SAS Institute Inc, Cary NC, USA). Participants’ attitudes toward sharing were characterized using descriptive statistics, and differences in attitude by age, sex, and self-rated health were assessed using chi-square tests of significance, *P* value < .05, and logistic regression on dichotomized values.

For the second two study groups, narrative data were collected in usability test and focus group discussion sessions. Open-ended responses to structured protocols were audio taped, transcribed, and analyzed for major thematic findings by a trained moderator and observer using previously reported methods [24]. For analysis of all narrative/text data, analysts worked independently with a shared rubric of major thematic codes to describe subject reports. Analysts read all narrative data independently to assign codes to text fragments and develop subsidiary coding schemes. Coding schemes and transcripts were worked iteratively and inductively to refine them and achieve consensus. Findings were reviewed and triangulated across the three assessment samples and activities—i.e., surveys conducted with demonstration evaluation participants, narrative and group interviews conducted with usability testers and community-based focus group participants—to build a comprehensive picture of issues related to attitudes toward sharing, conditions, and contexts. Differences across social groupings/factors including age, sex, social role/employment, and self-reported health status were assessed in analyses of survey data and these factors are commented on where available for analyses of qualitative reports from usability and focus group samples. Major constructs were operationally defined for thematic analysis. “Attitudes toward sharing of personal health information from PCHRs” was defined with respect to willingness and interest in making personal health information available to a health authority for purposes of monitoring, tracking, and needs assessment and preferences for sharing using opt-in or opt-out default designs. “Conditions” affecting sharing of health information from PCHRs for public health research were defined to include issues related to anonymity, privacy, confidentially, exclusive use for research, payment, and research intermediation. “Context” affecting willingness to share PCHR information was defined as the presence of a public health emergency.

We report major findings by thematic area for survey reports with demonstration evaluation participants in conjunction with findings from qualitative analyses, using quotes from focus group and user testing interviews to illustrate findings.

## Results

### Subject Characteristics

The total number of participants in all three subject groups was 181, and the majority, or 83% (151/181) were engaged in the nine-month demonstration of the live PCHR. Average age varied across the three groups (Table 1) and was youngest in the usability test group (45 years), reflecting an employee and student population, followed by the PCHR evaluation group (54 years) reflecting a community-based health maintenance organization population. Average age was greatest (71 years) among focus group subjects, a group drawn from a retiree and health advocacy mailing list. Females outnumbered males in each of the three groups, and most subjects self-reported that their race was white. Among the demonstration evaluation subjects, 70% (105/151) reported having good or excellent health. Demonstration evaluation subjects also reported high levels of education and moderately high levels of income. Data on income, household status, and education were not available for subjects in the focus group and the usability testing group.

**Table 1:** Characteristics of study samples by group

Sample group		PHCR Demonstration Evaluation	Focus Group Interviews	Usability Testing
		Number (%)	Number (%)	Number (%)
Total N		151	17	13
Mean age (SD)		54 (18)	71 (14)	45 (15)
Female sex		80 (53)	11 (65)	10 (77)
Lives with family		102 (68)	Not available	Not available
Self-rated health good to excellent		105 (70)	Not available	Not available
Education attained college or higher		136 (90)	Not available	Not available
Current student status		22 (15)	0 (0)	12 (92)
Income less than 100K		63 (42)	Not available	Not available
**Race**				
	White	130 (86)	14 (82)	9 (69)
	Asian	13 (9)	0 (0)	3 (23)
	American Indian	1 (0.7)	1 (8)	1 (8)
	African American	0 (0)	4 (24)	0 (0)

### Attitudes Toward Sharing for Health Research and Awareness of Sharing Options

Attitudes toward sharing health information for health research are reported for participants in the PCHR demonstration evaluation group (Table 2) and for usability and focus group participants using illustrative quotes (below). Of the participants in the PCHR demonstration, who were surveyed at the close of the demonstration, 91% (138/151) were agreeable to making their health information available for research (Table 2). Levels of endorsement were equally high for this group across sex, age, social role, and health status groupings.

**Table 2:** Attitudes of demonstration evaluation subjects toward sharing PCHR data for public health research

Measure		Total No.(%)	Female No. (%)	Male No.(%)	Age ≤ 50 No.(%)	Age > 50 No. (%)	Student No.(%)	Non student No.(%)	Poor Health^e^	Good Health^f^
Agreeable to sharing^d^		138 (91)	71 (89)	67 (94)	51 (91)	87 (92)	21 (95)	117 (91)	13 (93)	125 (91)
**Knows can share electronically**		61 (40)	31 (39)	30 (42)	23 (41)	38 (40)	13 (59)	48 (37)	6 (43)	55 (40)
	Of these, knows can share granularly	43 (71)	22 (71)	21 (70)	16 (70)	27 (71)	11 (85)	32 (67)	4 (67)	39 (71)
**Preferred model for sharing**										
	Sharing should be opt-in	89 (59)	50 (63)	39 (55)	31 (55)	58 (61)	8 (36) ^a^	81 (63) ^a^	81 (59)	8 (57)
	Sharing should be opt-out	54 (36)	27 (34)	27 (38)	22 (39)	32 (34)	12 (55) ^a^	42 (33) ^a^	5 (36)	49 (36)
**Conditions affecting sharing**	
	Anonymity: *increase* willingness	136 (90)	73 (91)	63 (89)	54 (96)	82 (86)	21 (95)	115 (89)	12 (86)	124 (91)
	Privacy not anonymity: *decrease* willingness	107 (71)	59 (74)	48 (68)	44 (79)	63 (66)	21 (95)	111 (86)	2 (14)	17 (12)
	Share request came from trusted intermediary: *increase* willingness	96 (64)	46 (58)	50 (70)	29 (52) ^a^	67 (71) ^a^	11 (50)	85 (66)	9 (64)	87 (64)
	Information only used for research: *increase* willingness	106 (70)	56 (70)	50 (70)	41 (73)	65 (68)	17 (77)	89 (69)	11 (79)	95 (69)
	Can view audit trail of access and sharing: *increase* willingness	119 (79)	61 (76)	58 (82)	47 (84)	72 (76)	18 (82)	101 (78)	12 (86)	107 (78)
	Payment for information: *increase* willingness	44 (29)	28 (35)	16 (23)	29 (52)^c^	15 (16)^c^	16 (73)^c^	28 (22)^c^	3 (21)	41 (30)
**Contexts affecting sharing**										
	Public health emergency: *increase* willingness	125 (83)	63 (79)	62 (87)	42 (75)^a^	83 (87)^a^	11 (50)^c^	114 (88)^c^	8 (57)^a^	117 (85)^a^

^a^ *P* < .05

^b^ *P* < .01

^c^ *P* < .001

^d^Includes very, moderately and somewhat agreeable

^e^Fair to poor self-rated health

^f^Good to excellent self-rated health

Only 61 of the 151 (40%) demonstration evaluation subjects reported knowing that they could provide others with read access to their PCHR and share its content electronically through the system’s sharing feature. Of these, 43 (71%) subjects replied that they understood that they could share portions of their record (granular sharing) rather than its entirety. No differences were observed in reported awareness across the various demographic and social groups. However, 54 out of 151 (36%) demonstration evaluation subjects thought sharing for public health research should be opt-out while 89 (59%) favored opt-in. Students were more likely than non-students to favor an opt-out default for sharing (unadjusted odds ration (OR) 2.89, 95% confidence interval [CI] 1.10-7.62, *P* = .03).

Among subjects in the usability testing and focus group samples, no clear preference for an opt-in/opt-out default for research was observed although some voiced an assumption that mandatory participation might eventuate. In either case, need for information and education was stipulated to advance the model:

Pretty soon no one’s gonna have any choice about it [opt-out or mandatory design] and the best thing you can do is to learn as much as you can and be prepared to maneuver through it so you can expose the least of your things that you can. Because they’re gonna… I think this is something that’s gonna be mandatory for everyone—you’re gonna have to.

Should be an opt-out, rather than opt-in. And should have a good educational piece that explains it.

Participating in public health research was contingent on receipt of an explanation of risks and benefits relating to sharing, including sharing genetic information:

I would have to know what is the worst-case scenario and what are the securities in place to prevent that; how likely is it that it will happen; what are the benefits. Knowing that you can opt-out or opt-in at any time. If there’s genetic data that has implications for family members, people should have informed consent about potential loss of privacy.

In principle, a fabulous idea. In this political environment, would not share anything! Certainly wouldn’t share with government. Opt-in would be ok, but no blanket permission.

Customization of access controls was described as a condition bearing on willingness to share personal health information from the PCHR and was framed by subjects in terms of content- driven restrictions that apply to topics, sections or domains in the record (ie, granularity) and time-limited restrictions (ie, temporality):

Would be willing to share data, as long as…could customize access [granularity]

Would be willing to share, as long as there’s appropriate privacy. Should be able to select what to leave out. [granularity]

Maybe you just get permission for 24 hours [temporality]

Do you give them permission…do you have to give them permission every time they go into it or is it forever? [temporality]

Which data exactly am I agreeing to share; which identifiers would be connected…how would it be used; is it on a one-time basis or recurrent; what kind of time limit [granularity and temporality]

### Conditions Affecting Engagement in Public Health Information Sharing

Almost all subjects in the demonstration evaluation group, 136 of 151 (90%), reported that guaranteeing conditions of strict anonymity would increase the likelihood they would share their health information for public health research. Findings were the same for focus group and usability test samples,

No name, no zip code, nothing.

They can’t know where you are.

A large majority of subjects in the demonstration evaluation group, 107 of 151 (71%), reported that guaranteeing privacy but not anonymity of shared health information would decrease their willingness to share. This perception was found in qualitative data also, where subjects in the focus group and usability testing group reported anticipating adverse consequences from disclosure of individually identifiable information:

An insurance company can take you off their rolls if they think you have too many illnesses.

…employers might not hire you if they think you’re sick.

[They might] deny you life insurance or something.

Of demonstration evaluation subjects, 119 of 151 (79%), responded that a system provision for viewing an audit trail of access to health information and a specific summary of shared data would increase their willingness to participate in sharing for public health research. Focus group and usability testing subjects also reported that an audit trail provision would increase trust and willingness to share data:

It’d be important to see who’s tried to get access to it. Same thing with financial information. Seems like the list is interminable after a while. It’s almost impossible to get off that list.

In qualitative interviews, subjects linked the availability of an audit trail with tight security controls as factors that would increase willingness to share their data, citing encryption of data as an example of such a security condition.

Most of the demonstration evaluation subjects, 106 of 151 (70%), reported that restricting the use of shared data to research would increase their willingness to share their data.

Also among this group, 96 of 151 (64%) reported that receipt of a request to share from a trusted intermediary (examples given to users were Children’s Hospital Boston and Harvard Medical School) would increase willingness to share. Persons older than 50 in this group were slightly more likely than younger persons to report that this condition would increase their willingness to share (unadjusted odds ratio [OR] 1.02, 95% confidence interval [CI] 1.00 - 1.04, *P* = .04). Engaging with a trusted intermediary around a request to share was similarly observed to facilitate willingness to share among focus group participants:

I don’t know. I’d have to know for sure that they are who they say they are. And, how would I find that out?

[You might not know] if it was a virus sent from your computer…

…if somebody puts out an all-encompassing email, saying “Would you let me look at your records?” how would I know who they are?

I think it would be useful. I’d do it. And I agree with [other member] that you’d have to have some way of knowing who you’re talking to, some phone number or something, some way to verify that the people are who they say the are.

If we knew Elissa, then maybe yes, why not? But without knowing her, just hearing that she’s from Harvard—well, Harvard’s a pretty big place. I don’t know.

[Would need to know]…that requester is part of an institution; that the requesters are doctors, not just random individuals.

Who would be the gatekeeper of that information? Who would tell that researcher that I had a certain illness?

A minority of demonstration evaluation subjects, 44 of 151 (29%), reported that payment for health information would increase willingness to share. Persons older than 50 were proportionately less likely to report that payment for health information would increase their willingness to share (unadjusted OR 0.94, 95% CI 0.91 - .96, *P* < .001); conversely, persons describing themselves as students reported that payment for data would increase their willingness to share (unadjusted OR 9.62, 95% CI 3.44 - 26.87, *P* < .001). For some usability testing group subjects, payment was perceived to increase safety/security of sharing when coupled with a trusted intermediary or requestor:

Would feel confident that data was safe if [he] was paid. Wouldn’t trust insurance company but would trust Harvard.

### Context of Public Health Emergency and Sharing for Public Health Research

Experience of a public health emergency was reported to increase willingness to share health information by 125 of 151 demonstration evaluation subjects (83%). Similar results were obtained in qualitative interviews among older, primarily retired, focus group participants and among usability testers who were employed and among whom qualified support was evident:

retiree I would be more likely to share during an epidemic/outbreak.

Would be more willing to share during epidemic/outbreak, but willing to share in general anyway.

Might be more willing to share in case of epidemic. [conditional on deidentified data]

employee In case of epidemic, before sharing, would want to know: What’s the scope of the epidemic; what type?

Experiencing a public health emergency was more likely to increase willingness to share among older users in the demonstration evaluation sample (unadjusted OR 1.03, 95% CI 1.01 - 1.05, *P* = 02). However, subjects in this sample who self-identified as students were proportionately less likely to report that a public health emergency would increase willingness to share data (unadjusted OR 0.13, 95% CI 0.05 - 0.36, *P* < .001). In this sample, persons with fair or poor self-rated health were less likely than subjects with good or excellent self-rated health to report that their willingness to share would increase during a public health emergency (unadjusted OR 0.61, 95% CI 0.38 - 0.97, *P* = .04).

## Discussion

### Principal Results

Across subject groups, regardless of level of exposure to personally controlled health record technology, sex, age, and social role (student or employee), we found high levels of willingness to share personal health information from a PCHR with public health agencies for purposes of disease monitoring, evaluation, and needs assessment. Pragmatism and altruism rather than naïveté seemed to characterize subject preferences and positions. A strong tendency was observed among subjects toward balancing privacy and safety concerns with the possibility of personal or societal gain stemming from public health research. While a greater preference for an opt-in versus opt-out default model was observed, the picture was mixed. Models of blanket information sharing for either default were not favored, and subjects recognized that regardless of the default model, successful approaches toward sharing would be contingent on ensuring clear understanding of risks and benefits associated with their actions. Time and content limitations to sharing were repeatedly suggested as important options for maintaining control over personal information. Other factors conditioning altruistic impulses were guarantees of anonymity, provisions for audit checks on record access and sharing patterns, intermediation from a trusted party, restrictions on use of information for research only and, for some subjects, payment/compensation for data.

Within these affirmative findings, it is notable that despite being sent messages about the feasibility and mechanics of sharing their PCHR electronically, proportionately few subjects, only 61 of 151 (40%) in the live PCHR demonstration understood they could share their record electronically. A substantial minority of these, 18 of 61 (29%), did not understand that they could share their data in a “granular fashion,” that is, selectively by topic, domain or section. These findings underscore the need for effective education and training on using this type of system to foster information flows of various types, recognizing that knowledge about sharing and attitudes toward doing so, including through granular controls, are likely to shift as the technology diffuses and opportunities to share increase.

Taken together, our findings suggest that longstanding concerns among technologists, advocates, and policy makers that consumer privacy concerns will undermine PCHR adoption, use, and sharing behaviors may not be born out if the sharing model and system is well-designed, well-executed, and well-explained [25-27]. As observed elsewhere [20], strong impulses toward information altruism auger well for new models of public health research that draw on PCHR data contributed by an engaged citizenry or patient populations.

### Limitations

Findings about willingness to share are promising, but it is worth noting that they reflect the views of a regionally sampled, nonrepresentative set of subjects and a specific form of personal health record. Inferences about broad population patterns and generic personal health records cannot be drawn. Findings reflect the attitudes of subjects with some of the earliest substantive experience with PCHRs, specifically with a live system that integrated medical records (redacted of clinician notes) with patient annotations about health-related behaviors, attitudes, and family/household contexts. Future testing with records that represent the full spectrum of clinical information including potentially sensitive information and notes is warranted as the technology continues to develop and diffuse. Additional research is needed to characterize attitudes toward sharing for research that reflects a more comprehensive spectrum of study conditions. It is likely that willingness to share will vary depending on type of data requested (genotypic, phenotypic, care system related, other), time horizon of investigation (cross-sectional vs longitudinal), study design (observational vs interventionist), purpose (discovery, commercial product development, care improvement, as well as surveillance) and by the affiliation and role of investigators (governmental, private, academic, other). All of these factors are important and deserve further study as does the role of incentives and feedback.

### Conclusion

Moving public health and medicine from a reactive to proactive stance with regard to detection and response to health problems may require seizing opportunities to engage consumers in health research using new approaches. There are clear advantages to exploring use of PCHRs as a vehicle for collecting health information germane to public health research: (1) the model may temper the one-way pull of data from subjects to investigators and authorities, providing a bridge for feedback and follow-up; (2) flexible cohort models may be facilitated given the dynamic nature of the system and the potential for ongoing ties to subjects; (3) emergency monitoring systems and rapid polling or surveillance of populations can reasonably be envisioned; and (4) linkage of phenotypic, service, and medical-biologic information in support of care improvement and discovery may be feasible. Success in these endeavors will depend on responding to preferences and conditions for fostering trust and maintaining ongoing research engagement. Such conditions include use of appropriate models for education and support of subjects and for obtaining their informed consent—a step that has proven elusive for electronic health record-based research [28]. In the rapidly evolving health information landscape, attention needs to be directed not only to defining preferences and principles for sharing, but also to defining the organizational and institutional mechanisms required for guarantees on safety and oversight.

## Abbreviations

HITECH: Health Information Technology for Economic and Clinical Health
HIPAA: Health Insurance Portability and Accountability Act
PCHR: personally controlled health record
